# ASO Author Reflections: Minimally Invasive Surgery for Prototypical Small Intestinal Neuroendocrine Tumors

**DOI:** 10.1245/s10434-024-15562-9

**Published:** 2024-05-31

**Authors:** Akitada Yogo, Eric K. Nakakura

**Affiliations:** 1 Division of Surgical Oncology, Section of Hepatopancreaticobiliary Surgery, Department of Surgery, University of California, San Francisco, San Francisco, CA USA; 2 Helen Diller Family Comprehensive Cancer Center, San Francisco, CA USA

## Past

Although minimally invasive surgery (MIS) is widely used for many gastrointestinal conditions, it is not routinely performed for the prototypical small intestinal neuroendocrine tumor (NET), i.e. an ileal NET (i-NET). For i-NETs, a pure laparoscopic approach is not adequate because patients often present with multifocal primary tumors and bulky lymphadenopathy/mesenteric mass that may extend to the root of the superior mesenteric vessels.^1,2^ We have previously shown that MIS utilizing a hand-port device allows surgeons to palpate the entire jejunum-ileum to identify multifocal primary tumors, and facilitates resection of the lymphadenopathy/mesenteric mass, with favorable short-term outcomes.^3,4^ However, the long-term outcomes of MIS for i-NETs are not known.

## Present

In our single-institution, retrospective cohort study of 168 patients,^5^ we compared the long-term overall survival (OS) of patients after MIS using a hand-port device with standard open resection. Open surgery was performed for bulky mesenteric mass or concurrent liver resection; otherwise, MIS was performed. Because the disease characteristics of the two cohorts were not balanced, we adjusted potential confounders by propensity score matching (PSM). After PSM, there was no significant difference in OS after a median follow-up of more than 4 years {median 99 months (95% confidence interval [CI] 91–not available [NA]) in MIS versus 103 months (95% CI 86–NA) in open surgery, *p* = 0.77; hazard ratio [HR] 0.87 (95% CI 0.33–2.2), *p* = 0.77} with a similar completion rate of mesenteric dissection (62% vs. 65%, *p* = 1).

## Future

MIS using a hand-port device is an alternative to open surgery for i-NETs, achieving similar short- and long-term oncological outcomes. Importantly, palpation is essential to identify multifocal primary tumors, and there should be no hesitation to convert to an open procedure to safely resect a bulky mesenteric mass. Our findings require corroboration by others given the variable experience in MIS worldwide.
